# Identification and Whole-Genome Sequence Analysis of Tembusu Virus GX2013G, Isolated from a Cherry Valley Duckling in Southern China

**DOI:** 10.1128/genomeA.00007-15

**Published:** 2015-02-19

**Authors:** Tingting Zeng, Zhixun Xie, Liji Xie, Xianwen Deng, Zhiqin Xie, Li Huang, Sisi Luo, Jiaoling Huang

**Affiliations:** Guangxi Key Laboratory of Animal Vaccines and Diagnostics, Guangxi Veterinary Research Institute, Nanning, Guangxi Province, China

## Abstract

A duck tembusu virus (DTMUV) was isolated from the brain of a Cherry Valley duckling that showed neurological signs by using a specific-pathogen-free chicken embryo. The isolate was named GX2013G (GenBank accession no. KM275941). The strain GX2013G was identified with reverse transcription-PCR (RT-PCR), and the amplicon was sequenced. The genome that was obtained is 10,990 nucleotides in length and contains a single open reading frame encoding a putative polyprotein of 3,425 amino acids. This study will advance the understanding of the epidemiology and molecular characteristics of tembusu virus (TMUV) in Guangxi and further studies of the mechanisms of virus replication and pathogenesis.

## GENOME ANNOUNCEMENT

Tembusu virus (TMUV) was isolated and identified for the first time in 2010 in the east of China (1). It was an extensive epidemic in the densest waterfowl breeding area of China, including Fujian (2), Shandong (3), Zhejiang (4), Jiangsu (5), and Guangxi (6). Almost all duck breeds were reported as having been infected by TMUV (3, 6–9); infections in geese and chickens were also reported (10, 11). Lying ducks showed a major decrease in egg production, high fever, loss of appetite, and neurological signs after infection. Morbidity was nearly 100%, but there was only a 0% to 12% mortality rate for the affected ducks. Ducklings could also be infected by TMUV, but were rarely reported.

In 2013, a flock of ducklings showed neurological signs and loss of appetite in a Cherry Valley duck farm in Guangxi. Encephaledema was observed, and the brains were collected to isolate viruses by using specific-pathogen-free (SPF) chicken embryos and identified by reverse transcription-PCR (RT-PCR). A strain of TMUV was isolated, and other pathogens which cause similar symptoms were ruled out (12–14). Thirteen pairs of primers were used to amplify the different regions of the strain GX2013G with an overlapping genome fragment covering each region. The 5′- and 3′-terminal sequences were determined by using a SMARTer rapid amplification of cDNA ends (RACE) cDNA amplification kit (Clonetech, Dalian, China). The amplified products were purified and cloned into pMD-18T vectors (Takara, Dalian, China) and sequenced (Invitrogen, Shanghai, China). Sequences were assembled by a Seqman program (Lasergene, Madison, USA) to produce the complete genome sequence of GX2013G. The full-length genome sequence of GX2013G was 10,990 nucleotides (nt) in length, with a typical flavivirus genome organization, and the 5′ and 3′ untranslated regions (UTR) were 94 and 618 nt, respectively. Additionally, the coding region of GX2013G included a single open reading frame (ORF) (10,278 nt) that encoded a polypeptide of 3,425 amino acids (aa), three structural proteins (capsid, prM, and envelope), and seven nonstructural proteins (NS1, NS2A, NS2B, NS3, NS4A, NS4B, and NS5).

Compared to genome sequences of previously isolated TMUVs from different species of ducks, geese, and chickens in various areas of China, there were 96.5% to 97.5% homology at the nucleotide level and 99.7% homology with GX2013H isolated in Guangxi (6). A phylogenetic tree based on the whole sequence showed that GX2013G was in a single clade with GX2013H, whereas other strains were in a different clade. It was demonstrated that TMUVs in China had conservative nucleic acid levels and the most homology in the area.

By predicting the potential glycosylation sites, we found 13 glycosylation sites in six viral proteins: 2 glycosylation sites in prM, 1 in E, 3 in NS1, 1 in NS2A, 3 in NS4B, and 3 in NS5. The numbers and positions of glycosylation sites in each protein were the same as GX2013H.

In conclusion, this study of the whole-genome sequence of TMUV provides further information about the epidemiology and evolution of TMUV and may help elucidate mechanisms of virus replication and pathogenesis.

### Nucleotide sequence accession number.

The complete genome sequence of the duck tembusu virus isolate has been deposited in GenBank under the accession no. KM275941.
